# Prognostic Significance of Nephrotic-Range Proteinuria in Paediatric Acute Glomerulonephritis

**DOI:** 10.7759/cureus.111283

**Published:** 2026-06-22

**Authors:** Atanka N Samal, Lipsa Priyadarshini, Sumanta Panigrahi

**Affiliations:** 1 Department of Paediatrics, Maharaja Jajati Keshari Medical College and Hospital, Jajpur, IND; 2 Department of Paediatrics, Sardar Vallabhbhai Patel Post Graduate Institute of Paediatrics, Srirama Chandra Bhanja Medical College and Hospital, Cuttack, IND; 3 Department of Paediatrics, Government Medical College and Hospital, Kandhamal, Phulbani, IND

**Keywords:** acute glomerulonephritis, children, follow-up, nephritic-nephrotic syndrome, prognosis

## Abstract

Background: Acute glomerulonephritis (AGN) in paediatric patients typically presents with haematuria, oedema, hypertension, oliguria, and a decline in renal function attributable to immune‑mediated glomerular injury. A subset of patients presents with nephrotic-range proteinuria at onset, termed nephritic-nephrotic syndrome, the prognostic implications of which remain underexplored.

Methods: A prospective observational study was conducted in the departments of paediatrics and paediatric nephrology at a tertiary care centre in Odisha over two years. Children aged 1-14 years with a clinical diagnosis of AGN were enrolled and evaluated for demographic, clinical, and laboratory parameters. Patients with nephrotic-range proteinuria at presentation were analysed as a distinct subgroup. Clinical course, complications, and outcomes were compared between nephritic-nephrotic and classical nephritic presentations.

Results: Seventy children were included (53 males, 17 females); the majority were aged 5-10 years. Facial puffiness (100%), haematuria (68.6%), and pedal oedema (64.3%) were the most frequent presenting features. A preceding infection was documented in 85.7%, commonly pharyngitis (58.6%) or skin infection (27.1%). Nephrotic-range proteinuria was observed in 15 patients (21%). Complications on admission were more frequent in this subgroup, including hypertensive encephalopathy (60% vs. 9.1%), congestive cardiac failure (60% vs. 16.4%), and acute kidney injury (60%% vs. 21.8%). There were many signs and symptoms overlapping in both hypertensive encephalopathy and congestive cardiac failure in nephrotic-range proteinuria patients. At the three-month follow-up, children with nephrotic-range proteinuria were found to develop persistent hypertension.

Conclusion: Paediatric AGN with nephrotic-range proteinuria represents a high-risk phenotype associated with a more severe clinical course, greater complication rates, and may have adverse long-term renal outcomes. Early recognition and close follow-up of this subgroup are essential for timely intervention and improved prognosis.

## Introduction

Acute glomerulonephritis (AGN) usually begins abruptly and may present with haematuria, oedema, hypertension, and oliguria. It is often accompanied by reduced glomerular filtration, sodium and fluid retention, and features of circulatory congestion. When an infectious organism is the precipitant, the condition is termed acute post‑infectious glomerulonephritis (APIGN) and has been associated with a range of pathogens, including bacteria [1], viruses, parasites, and mycoplasma. APIGN represents an immune‑mediated glomerular injury marked by intraglomerular inflammation and cellular proliferation driven by the host response to these infectious agents [2].

Post‑streptococcal glomerulonephritis (PSGN), typically following throat infection with nephritogenic group A beta‑hemolytic *Streptococcus*, constitutes the commonest subtype of APIGN and a major contributor to acute kidney injury [3,4]. Although many cases are subclinical and the exact incidence is difficult to determine, APIGN remains an important public‑health problem, particularly in low‑ and middle‑income countries [3]. The global burden is concentrated in developing regions, while incidence has declined in industrialised nations. Epidemiological estimates indicate hundreds of thousands of cases annually worldwide, with the majority occurring in developing countries [4]; reported incidence rates contrast sharply between less developed and industrialised settings [5].

Clinically, PSGN and related APIGN syndromes commonly present with sudden microscopic or gross haematuria, oedema, hypertension, oliguria, azotaemia, and variable proteinuria [4]. Protein excretion in PSGN is generally below the nephrotic range (commonly <1 g/m^2^/day), and oedema primarily reflects sodium and water retention. By contrast, clinically, nephrotic syndrome is typified by heavy proteinuria, consequent hypoalbuminemia, elevated plasma lipids, and widespread fluid accumulation [6]. Some patients exhibit a mixed nephritic-nephrotic picture, which typically indicates mesangial and membranous involvement [7]; those with nephrotic‑range proteinuria in the context of PSGN tend to have a worse prognosis, with greater risk of persistent hypertension and progression to chronic glomerulonephritis.

The aim of this study was to evaluate the clinico-laboratory profile and clinical presentation of AGN, with special emphasis on patients presenting with nephrotic-range proteinuria at onset (nephritic-nephrotic syndrome), to assess the laboratory parameters used for diagnosis and to examine the course of the illness in this part of the country.

## Materials and methods

We conducted a prospective study in the Department of Paediatrics and the Department of Paediatric Nephrology at Sardar Vallabhbhai Patel Post Graduate Institute of Paediatrics (SVPPGIP) and Srirama Chandra Bhanja Medical College and Hospital (SCBMCH), Cuttack, over a period of two years from July 2022 to July 2024 after obtaining ethical approval from the Institutional Ethical Committee. SVPPGIP is a postgraduate institute of paediatrics, which is associated with SCBMCH, Cuttack. It is a tertiary care hospital in the state of Odisha, India.

Aims and objectives

Primary Objective

To compare outcomes between children with and without nephrotic‑range proteinuria at presentation.

Secondary Objective

To assess whether nephrotic‑range proteinuria serves as a predictor of adverse outcomes, including hypertension, acute kidney injury, and progression to chronic glomerulonephritis.

Inclusion criteria

Inclusion criteria included the following: (1) children aged between one year and 14 years; (2) urinary findings of gross or microscopic haematuria; (3) urinary findings of nephrotic-range proteinuria defined as >40 mg/m²/hr or spot urine protein/creatinine ratio (spot PCR) >2; (4) sudden onset of oliguria and oedema; (5) minimum follow‑up of three months from enrolment.

Exclusion criteria

The children meeting the following criteria were excluded from the study: (1) previously diagnosed case of nephrotic syndrome; (2) previous history of any renal disease, hypertension, chronic liver disease, any protein-losing enteropathy or malabsorption, or malnutrition; (3) other causes of haematuria and hypertension excluded clinically and with appropriate diagnostic investigations; (4) other causes of haemoglobinuria and other causes of high coloured urine.

Sample size

The study sample was selected using consecutive sampling, where every patient admitted during the study period who satisfied the inclusion criteria and whose guardian gave informed consent was included. Missing data were minimised by prospective design, and cases with incomplete follow‑up were excluded. The parents/guardians of the patients were informed about the aim, method, and duration of the study. Cases were then enrolled after the consent of the parents/guardians. Data variables, including age, sex, socioeconomic status, and predisposing risk factors such as upper respiratory tract infection (URTI) and pyoderma, were then collected in a prescribed proforma prepared for this study.

A detailed history was obtained, documenting onset and duration of haematuria, peri‑orbital oedema, oliguria, and other associated symptoms. All participants underwent a comprehensive physical examination and relevant laboratory investigations, including complete blood count, serum electrolytes, blood urea, serum creatinine, urinalysis, and urine culture with sensitivity testing. Radiological evaluation was done whenever required.

Diagnostic criteria

Acute kidney injury was defined according to Kidney Disease: Improving Global Outcomes (KDIGO) guidelines [8]. Congestive cardiac failure [9] and hypertensive encephalopathy [10,11] were diagnosed using standard paediatric clinical criteria.

Laboratory cut‑offs were as follows: antistreptolysin O (ASO) titres >200 IU/ml were considered positive; complement C3 <90 mg/dl was considered low.

For blood pressure classification, hypertension was defined according to age‑appropriate paediatric norms.

Haemoglobin level of 10-10.9 g/dL was defined as mild anaemia, 7-9.9 g/dL as moderate anaemia, and <7 g/dL as severe anaemia [12].

Follow‑up

After discharge, patients were reviewed at three‑month intervals in paediatric and nephrology OPDs. At each visit, blood pressure, urine protein, and renal function were assessed. Persistent hypertension/proteinuria and progression to chronic glomerulonephritis were defined using standard paediatric nephrology criteria. Several patients were followed up for six months. Cases with incomplete follow‑up were excluded.

Statistical analyses

Statistical analyses were performed using IBM SPSS version 22 (IBM Corp., Armonk, NY). Categorical variables are expressed as frequencies and percentages. Associations between categorical variables were assessed with the chi-square test. Continuous variables are reported as mean ± SD. Paired comparisons (baseline versus follow‑up) were evaluated with the paired t‑test, and comparisons between two independent groups were made using the independent t‑test. A significance threshold of p ≤ 0.05 was used.

## Results

Of the 70 children enrolled in the study, 53 (75.7%) were male, and 17 (24.3%) were female. A total of nine (12.8%), 45 (64.3%), and 16 (22.8%) children were in the age group of one year to four years and 11 months, five years to nine years and 11 months, and 10 years to 14 years, respectively. Many participants fell within the age group of five years to nine years and 11 months. The most common symptom was facial puffiness (70, 100%), followed by haematuria (48, 68.6%), pedal oedema (45, 64.3%), oliguria (44, 62.9%), headache (28, 40%), and altered sensorium (6, 8.5%), presenting with hypertensive encephalopathy. A total of 60 (85.7%) children had a history of preceding infection, such as sore throat (41, 58.6%) and skin infection (19, 27.1%).

At baseline, the cohort (n = 70) demonstrated several notable laboratory abnormalities. Hyponatremia (<130 mmol/L) was present in 21.4%, while the majority had sodium within the reference range (78.6%). Hyperkalaemia (>5.5 mmol/L) occurred in 31.4%, and hypocalcaemia (<1.2 mmol/L) was highly prevalent (82.9%). Anaemia was common, predominantly mild (65.7%), with an additional 24.3% showing moderate anaemia (haemoglobin = 7-10.9 g/dl). Inflammatory markers were positive in a minority (CRP positive = 24.3%). All patients had low complement C3 (<60 mg/dl), and ASO positivity (>200 I.U/ml) was found in 77.1%, while antinuclear antibodies (ANA) were negative in all cases (Table 1).

**Table 1:** Baseline laboratory parameters of the study participants (n = 70). Gm: gram; m^2^: meter square; %: percentage; g/dl: gram per decilitre; mg/dl: milligram per decilitre; mmol/lit: millimoles per litre; TLC/mm^3^: total leucocyte counts per millimetre cube; CRP-C: reactive protein; ASO: antistreptolysin O; ANA: antinuclear antibodies; Hb: haemoglobin; C3: compliment C3; IU: international unit; ml: millilitre.

Lab parameters	Values	Frequency (%)
Sodium (mmol/lit)	<130	15 (21.4%
	130-150	55 (78.6%)
	>150	0 (0.0%)
Potassium (mmol/lit)	<3.5	0 (0.0%)
	3.5-5.5	48 (68.6%)
	>5.5	22 (31.4%)
Calcium (mmol/lit)	<1.2	58 (82.9%)
	1.2-1.38	8 (11.4%)
	>1.38	4 (5.7%)
Cholesterol (mg/dl)	<130	9 (12.9%)
	130-200	46 (65.7%)
	>200	15 (21.4%)
Total protein (gm/dl)	<6	44 (62.9%)
	>6	26 (37.1%)
Albumin (gm/dl)	<3.5	52 (74.3%)
	>3.5	18 (25.7%)
Hb (g/dl)	Severe anaemia (<7)	0 (0.0%)
	Moderate anaemia (7-9.9)	17 (24.3%)
	Mild anaemia (10-10.9)	46 (65.7%)
	No anaemia (>11)	7 (10.0%)
TLC/mm^3^	<5000	18 (25.7%)
	5000-10000	43 (61.4%)
	>10000	9 (12.9%)
CRP	Positive	17 (24.3%)
	Negative	53 (75.7%)
C3 (mg/dl)	<90	70 (100.0%)
	>90	0 (0.0%)
ASO (>200 IU/ml)	Positive	54 (77.1%)
	Negative	16 (22.9%)
ANA	Negative	70 (100.0%)
Spot urinary protein/creatine ratio	<2	55 (78.6%)
	>2	15 (21.4%)

The clinical complications differed markedly by proteinuria status. In patients with a nephrotic range of proteinuria (n = 15), there were higher rates of complications like hypertensive encephalopathy (60% vs. 9.1%, p < 0.001) and congestive cardiac failure (60% vs. 16.4%, p < 0.001) during admission, which was statistically significant. Similarly, 60% of patients had acute kidney injury in the nephrotic group of proteinuria compared to 21.8% in the non-nephrotic group of proteinuria, with a p-value of 0.004, which was statistically significant. The patients in the nephrogenic-proteinuria group presented with hypertensive encephalopathy, which later led to congestive cardiac failure. There was an overlap of multiple complications at the time of admission in patients with a nephrotic range of proteinuria (Table 2). So, there were more complications during the admission of patients with heavy proteinuria.

**Table 2:** Clinical complications among AGN cases with/without nephrotic-range proteinuria. AGN: acute glomerulonephritis; CCF: congestive cardiac failure; AKI: acute kidney injury; %: percentage. * Degree of freedom.

Complications	AGN with nephrotic-range proteinuria (n = 15), Frequency (%)	AGN with non-nephrotic-range proteinuria (n = 55), Frequency (%)	Chi-square (x^2^), df*	P-value
CCF	9 (60.0%)	9 (16.4%)	X^2 ^= 10.92, df = 1	0.001
Hypertensive encephalopathy	9 (60.0%)	5 (9.1%)	X^2 ^= 17.65, df = 1	<0.001
AKI	9 (60.0%)	12 (21.8%)	X^2 ^= 8.12, df = 1	0.004

Overall renal and urinary indices improved at follow‑up. Mean urine red blood cells/high power field (RBC/HPF) fell from 56.99 ± 8.1 at baseline to 9.27 ± 4.06 (p < 0.001). Serum urea decreased from 51.87 ± 19.2 to 41.31 ± 11.1 (p < 0.001), and serum creatinine declined from 1.30 ± 1.00 to 0.75 ± 0.48 (p < 0.001). Proteinuria grade shifted towards lower categories: the proportion with trace/1+ proteinuria rose from 48.6% to 82.9%, while those with 2+-4+ fell from 51.4% to 17.1% (p < 0.001). The urine protein/creatinine ratio improved, with the proportion >2 decreasing from 21.4% to 8.6% (p = 0.03) (Table 3).

**Table 3:** Comparison of baseline and follow-up indicators (n = 70). RBC: red blood cells; HPF: high power field; SD: standard deviation; %: percentage; CI: confidence interval. * Degree of freedom.

Variables	Baseline (Mean ± SD) (95% CI)	Follow-up (Mean ± SD) (95% CI)	Paired t-test value df*	p-value
Urine RBC/HPF	56.99 ± 8.1 (55-58.8)	9.27 ± 4.06 (8.3-10.2)	t = 45.2, df = 69	<0.001
Serum urea	51.87 ± 19.2 (47.3-56.3)	41.31 ± 11.1 (38.7-43.9)	t = 4.92, df = 69	<0.001
Serum creatinine	1.3 ± 1.003 (1-1.5)	0.75 ± 0.48 (0.63-0.86)	t = 4.39, df = 69	<0.001
Proteinuria	Baseline frequency (%)	Follow-up frequency (%)	Chi-square & df*	p-value
Urine protein, trace & 1+	34 (48.6%)	58 (82.9%)	X^2 ^= 15.72	<0.001
Urine protein, 2+,3+,4+	36 (51.4%)	12 (17.1%)	X^2 ^= 15.72, df = 1	<0.001
Urine protein/creatinine ratio <2	55 (78.6%)	64 (91.4%)	X^2 ^= 4.32, df = 1	0.03
Urine protein/creatinine ratio >2	15 (21.4%)	6 (8.6%)	X^2 ^= 4.32, df = 1	0.03

Blood pressure and renal function showed incomplete recovery in the nephrotic-range group. Mean systolic blood pressure decreased from 143.6 ± 9.8 mmHg to 129.6 ± 9.2 mmHg (p < 0.001) and diastolic from 88.4 ± 4.2 mmHg to 84.4 ± 4.9 mmHg (p = 0.05). Haematuria improved markedly (mean RBC/HPF = 59.8 to 12.9, p < 0.001). Severe proteinuria (3+-4+) declined from 100% at baseline to 33.3% at follow‑up (p < 0.001). Serum creatinine in this subgroup fell from 2.75 ± 0.6 mg/dl to 1.32 ± 0.42 mg/dl (p < 0.001), though values remained higher than in the non‑nephrotic group (Table 4).

**Table 4:** Blood pressure and laboratory parameters in patients with AGN and nephrotic-range proteinuria (n = 15). AGN: acute glomerulonephritis; mmHg: millimetre of mercury; RBC: red blood cells; HPF: high power field; SD: standard deviation; %: percentage; CI: confidence interval. * Degree of freedom.

Blood pressure	Baseline (Mean ± SD) (95% CI)	Follow-up (Mean ± SD) (95% CI)	Paired t-test value, df*	p-value
Systolic blood pressure (mmHg)	143.6 ± 9.8 (138.6-148.5)	129.6 ± 9.2 (121.9-131.9)	4.52, df = 14	<0.001
Diastolic blood pressure (mmHg)	88.4 ± 4.2 (86.27-90.52)	84.4 ± 4.9 (81.9-86.8)	2.12, df = 14	0.05
Urine RBC/HPF	59.80 ± 6.9 (56.29-63.3)	12.87 ± 17.29 (4.12-21.61)	9.45, df = 14	<0.001
Serum creatinine <1.24	2.75 ± 0.6 (2.4-3)	1.32 ± 0.42 (1.1-1.5)	8.12, df = 14	<0.001
Proteinuria	Baseline frequency (%)	Follow-up frequency (%)	Chi-square (X^2^), *df	p-value
Urine protein, 1+, 2+	0 (0.0%)	10 (66.7%)	X^2 ^= 11.12, df = 1	<0.001
Urine protein, 3+, 4+	15 (100%)	5 (33.3%)	X^2 ^= 15, df = 1	<0.001

Patients without nephrotic‑range proteinuria also improved, with larger absolute reductions in blood pressure. Mean systolic blood pressure fell from 130.3 ± 7.8 mmHg to 106.7 ± 10.3 mmHg (p < 0.001) and diastolic blood pressure from 84.2 ± 4.7 mmHg to 69.5 ± 9.1 mmHg (p < 0.001). At follow‑up, between‑group comparisons showed that the nephrotic group had significantly higher systolic and diastolic pressures than the non‑nephrotic group (systolic = 129.6 vs. 106.7 mmHg, diastolic = 84.4 vs. 69.5 mmHg, both p < 0.001) (Table 5).

**Table 5:** Blood pressure among patients with AGN without nephrotic-range proteinuria. AGN: acute glomerulonephritis; mmHg: millimetre of mercury; SD: standard deviation; %: percentage; CI: confidence interval. * Degree of freedom.

Blood pressure	Baseline (Mean ± SD) (95% CI)	Follow-up (Mean ± SD) (95% CI)	Paired t-test value, df*	p-value
Systolic blood pressure (mmHg)	130.3 ± 7.8 (128.2-132.3)	106.7 ± 10.3 (103.9-109.4)	15.42, df = 69	<0.001
Diastolic blood pressure (mmHg)	84.2 ± 4.7 (82.9-85.4)	69.5 ± 9.1 (67-71-9)	12.87, df = 69	<0.001

## Discussion

A total of 70 children were included, with the majority aged five to 10 years (64.3%). The mean age was 8.9 years. In a study by Sepahi et al., the mean age was 8.2 years [13]. Malla et al. reported that 95.2% of children were older than five years [14]. Males constituted 75.71% of cases, yielding a male-to-female ratio of 3.1:1. Becquet et al. reported a similar ratio of approximately 2.2:1 [15].

Clinical presentations were diverse. Facial puffiness was universal, followed by haematuria (68.6%), pedal oedema (64.3%), oliguria (62.9%), headache, and altered sensorium. These findings align with those reported by Yadav et al. [16]. However, symptom prevalence varies by cohort. Singh et al. reported pedal oedema as the most common symptom (91%) [17].

Of the 70 children, 15 (21.4%) had nephrotic-range proteinuria. Quantification was performed using spot urine protein/creatinine ratio (PCR), a validated paediatric method. Complications at admission were monitored and documented during hospitalisation. Most patients were hypertensive at presentation, and baseline blood pressure was higher in the nephrotic-range proteinuria group. Blood pressure decreased significantly during follow-up in both groups, though it remained higher in those with nephrotic-range proteinuria compared with non-nephrotic counterparts. Becquet et al. reported that no patients were hypertensive by five months post-illness, with a mean duration of hypertension of approximately three weeks [15].

Immunologic assessments showed hypocomplementemia in all patients, elevated ASO (ASO > 200 IU/ml) titres in 77%, and negative ANA in all. ASO negativity (ASO < 200 IU/ml) may reflect infections by non-streptococcal organisms, pyoderma, timing of sample collection, or variability in immune response. Becquet et al. reported 100% haematuria at presentation, proteinuria in 98%, elevated ASO in 76.3%, decreased C3 in 90%, and azotaemia in 70% [15]. Albar et al. found microscopic haematuria in 99.3%, proteinuria in 98.5%, elevated ASO in 66.6%, hypocomplementemia in 60.4%, and azotaemia in 19.1% [18].

Serum urea and creatinine were frequently elevated initially, with significant improvement during follow-up. Patients with nephrotic-range proteinuria showed more persistent elevations compared with others, suggesting a correlation between proteinuria severity and renal impairment. Becquet et al. reported a mean persistence of proteinuria of approximately four months, and Gunasekaran et al. noted proteinuria at six months in 7.7% of cases [15,19].

Laboratory findings were consistent across the cohort: microscopic haematuria and proteinuria were universal. Nephrotic-range proteinuria was observed initially in 21.4% but declined to 8.6% during follow-up, indicating recovery. Chugh et al. found 14% with nephrotic-range proteinuria at onset, while Poudel et al. reported 6.67% [20,21].

The chronology of complications at admission was hypertensive encephalopathy, followed by congestive cardiac failure and acute kidney injury. Complications were more frequently observed in patients with nephrotic-range proteinuria than in those with non-nephrotic-range proteinuria. Hypertensive encephalopathy (60% vs. 9.1%, p < 0.001), congestive cardiac failure (60% vs. 21.8%, p < 0.001), and acute kidney injury(60% vs. 21.8%, p = 0.004) were more frequent in nephrotic range of proteinuria patients. Similar associations were reported by Ge et al. in a 2020 cohort from China [22].

Limitations

This study has certain limitations that warrant consideration. The relatively small sample size may restrict the statistical power and generalizability of the findings, and the follow‑up duration was limited to three months for most patients, with only a subset reaching six months, which does not allow full assessment of long‑term renal outcomes. Multivariable regression analysis was not performed, so potential confounding factors influencing incomplete recovery could not be fully adjusted for. As the study was conducted in a single tertiary care centre, external validity may be limited, and some laboratory and radiological investigations were performed based on clinical indication rather than uniformly across all participants, introducing variability. In addition, a small proportion of patients did not complete the minimum follow‑up period and were excluded, which may have introduced bias. Despite these constraints, the study provides valuable insights into the clinical spectrum and short‑term outcomes of AGN in children, highlighting predictors of incomplete recovery and areas for future research.

## Conclusions

This study offers critical insights into the clinical spectrum of AGN in children, highlighting the distinct challenges posed by cases presenting with nephrotic-range proteinuria. Children with nephritic-nephrotic syndrome exhibited a significantly more severe disease trajectory, marked by increased incidence of hypertension and acute kidney injury. In contrast, those without nephrotic-range proteinuria generally experienced milder courses and more favourable outcomes. As there is a lack of long-term follow-up, in a short-term follow-up of three months, prognostic importance could not be established. However, there was an association between severe complications at admission and patients in the nephrotic-range proteinuria group, necessitating vigilant clinical surveillance during admission and follow-up. Early identification and aggressive management of hypertension, congestive cardiac failure, and acute kidney injury are required to avoid worse outcomes. Structured long-term follow-up is imperative to mitigate renal morbidity and improve long-term outcomes in this vulnerable paediatric population.

## References

[1] Moroni G, Claudio P (2009). Acute post-infectious glomerulonephritis. Treatment of Primary Glomerulonephritis, 2nd Edition.

[2] Stratta P, Musetti C, Barreca A, Mazzucco G (2014). New trends of an old disease: the acute post infectious glomerulonephritis at the beginning of the new millenium. J Nephrol.

[3] Kanjanabuch T, Kittikowit W, Eiam-Ong S (2009). An update on acute postinfectious glomerulonephritis worldwide. Nat Rev Nephrol.

[4] Eison TM, Ault BH, Jones DP, Chesney RW, Wyatt RJ (2011). Post-streptococcal acute glomerulonephritis in children: clinical features and pathogenesis. Pediatr Nephrol.

[5] Alper C (2012). The kidney. Robbins and Cotran Pathologic Basis of Disease, 8th Edition.

[6] Ponticelli C, Moroni G (2023). Nephrotic syndrome: pathophysiology and consequences. J Nephrol.

[7] Barrios JE, Duran Botello C, González Velásquez T (2012). Nephrotic syndrome with a nephritic component associated with toxoplasmosis in an immunocompetent young man. Colomb Med (Cali).

[8] Kidney Disease: Improving Global Outcomes (KDIGO) Acute Kidney Injury Work Group (2012). KDIGO clinical practice guideline for acute kidney injury. Kidney Int Suppl.

[9] Price JF (2019). Congestive heart failure in children. Pediatr Rev.

[10] Ahn CH, Han SA, Kong YH, Kim SJ (2017). Clinical characteristics of hypertensive encephalopathy in pediatric patients. Korean J Pediatr.

[11] Wiraboonchai B, Khongkhatithum C, Nimkarn N, Chantarogh S, Saisawat P, Tangnararatchakit K, Pirojsakul K (2025). Clinical characteristics and outcomes of children with hypertensive encephalopathy. BMC Pediatr.

[12] Khurana R, Kanvinde P, Mudaliar S (2024). Revised WHO guidelines on hemoglobin cutoffs to define anemia in individuals and populations. Indian Pediatr.

[13] Sepahi MA, Shajari A, Shakiba M, Shooshtary FK, Salimi MH (2011). Acute glomerulonephritis: a 7 years follow up of children in center of Iran. Acta Med Iran.

[14] Malla K, Sarma MS, Malla T, Thaplial A (2008). Varied presentations of acute glomerulonephritis in children: single centre experience from a developing country. Sultan Qaboos Univ Med J.

[15] Becquet O, Pasche J, Gatti H, Chenel C, Abély M, Morville P, Pietrement C (2010). Acute post-streptococcal glomerulonephritis in children of French Polynesia: a 3-year retrospective study. Pediatr Nephrol.

[16] Yadav SP, Shah GS, Mishra OP, Baral N (2016). Pattern of renal diseases in children: a developing country experience. Saudi J Kidney Dis Transpl.

[17] Singh M, Azizi E, Qureshi MA, Arya LS (1984). Clinical profile of acute glomerulonephritis in children. Indian J Pediatr.

[18] Albar H, Rauf S (2016). The profile of acute glomerulonephritis among Indonesian children. Paediatr Indones.

[19] Gunasekaran K, Krishnamurthy S, Mahadevan S, Harish BN, Kumar AP (2015). Clinical characteristics and outcome of post-infectious glomerulonephritis in children in southern India: a prospective study. Indian J Pediatr.

[20] Chugh KS, Malhotra HS, Sakhuja V (1987). Progression to end stage renal disease in post-streptococcal glomerulonephritis (PSGN)—Chandigarh study. Int J Artif Organs.

[21] Poudel DR, Basnet S, Gami FC (2014). Postinfective glomerulonephritis (PIGN) in children attending a tertiary care centre in Nepal. J Nepal Paediatr Soc.

[22] Ge H, Wang X, Deng T, Deng X, Mao H, Yuan Q, Xiao X (2020). Clinical characteristics of acute glomerulonephritis with presentation of nephrotic syndrome at onset in children. Int Immunopharmacol.

